# FGF signalling inhibition in ESCs drives rapid genome-wide demethylation to the epigenetic ground state of pluripotency

**DOI:** 10.1186/1868-7083-5-S1-S2

**Published:** 2013-08-19

**Authors:** Gabriella Ficz, Timothy A Hore, Fatima Santos, Heather J Lee, Wendy Dean, Julia Arand, Felix Krueger, David Oxley, Yu-Lee Paul, Jörn Walter, Simon J Cook, Simon Andrews, Miguel R Branco, Wolf Reik

**Affiliations:** 1 Epigenetics Programme, The Babraham Institute, Cambridge, CB22 3AT, UK; 2 Department of Biological Sciences, Institute of Genetics/Epigenetics, University of Saarland, Saarbrüken, Germany; 3 Bioinformatics Group, Babraham Institute, Cambridge CB22 3AT, UK; 4 Proteomics Research Group, Babraham Institute, Cambridge CB22 3AT, UK; 5 Signalling Programme, The Babraham Institute, Cambridge, CB22 3AT, UK; 6 Centre for Trophoblast Research, University of Cambridge, Cambridge CB2 3EG, UK; 7 Wellcome Trust Sanger Institute, Hinxton CB10 1SA, UK

## 

Genome-wide erasure of DNA methylation takes place in primordial germ cells (PGCs) and early embryos and is linked with pluripotency. Inhibition of Erk1/2 and Gsk3β signalling in mouse embryonic stem cells (ESCs) by small molecule inhibitors (called 2i) has recently been shown to induce hypomethylation. We show by whole-genome bisulphite sequencing that 2i induces rapid and genome-wide demethylation on a scale and pattern similar to that in migratory PGCs and early embryos. Major satellites, intracisternal A particles (IAPs) and imprinted genes remain relatively resistant to erasure. Demethylation involves oxidation of 5-methylcytosine (5mC) to 5-hydroxymethylcytosine (5hmC), impaired maintenance of 5mC and 5hmC and repression of the *de novo* methyltransferases (Dnmt3a, Dnmt3b) and Dnmt3L. We identify a Prdm14 and Nanog binding *cis*-acting regulatory region in *Dnmt3b* that is highly responsive to signalling. These insights provide a novel framework for understanding how signalling pathways regulate reprogramming to an epigenetic ground state of pluripotency.

